# Functional profile of perilesional gray matter in focal cortical dysplasia: an fMRI study

**DOI:** 10.3389/fnins.2024.1286302

**Published:** 2024-01-22

**Authors:** Bo Jin, Jiahui Xu, Chao Wang, Shan Wang, Hong Li, Cong Chen, Linqi Ye, Chenmin He, Hui Cheng, Lisan Zhang, Shuang Wang, Jin Wang, Thandar Aung

**Affiliations:** ^1^Department of Neurology, Sir Run Run Shaw Hospital, School of Medicine, Zhejiang University, Hangzhou, China; ^2^Department of Radiology, Second Affiliated Hospital, School of Medicine, Zhejiang University, Hangzhou, China; ^3^Department of Neurology, Epilepsy Center, Second Affiliated Hospital, School of Medicine, Zhejiang University, Hangzhou, China; ^4^Department of Neurology, Epilepsy Center, University of Pittsburgh Medical Center, Pittsburgh, PA, United States

**Keywords:** epilepsy, focal cortical dysplasia, antiseizure medication, gray matter, functional MRI

## Abstract

**Objectives:**

We aim to investigate the functional profiles of perilesional gray matter (GM) in epileptic patients with focal cortical dysplasia (FCD) and to correlate these profiles with FCD II subtypes, surgical outcomes, and different antiseizure medications (ASMs) treatment response patterns.

**Methods:**

Nine patients with drug-responsive epilepsy and 30 patients with drug-resistant epilepsy (11 were histologically confirmed FCD type IIa, 19 were FCD type IIb) were included. Individual-specific perilesional GM and contralateral homotopic GM layer masks were generated. These masks underwent a two-voxel (2 mm) dilation from the FCD lesion and contralateral homotopic region, resulting in 10 GM layers (20 mm). Layer 1, the innermost, progressed to Layer 10, the outermost. Amplitude of low-frequency fluctuations (ALFF) and regional homogeneity (ReHo) analyses were conducted to assess the functional characteristics of ipsilateral perilesional GM and contralateral homotopic GM.

**Results:**

Compared to the contralateral homotopic GM, a significant reduction of ALFF was detected at ipsilateral perilesional GM layer 1 to 6 in FCD type IIa (after Bonferroni correction *p* < 0.005, paired *t*-test), whereas a significant decrease was observed at ipsilateral perilesional GM layer 1 to 2 in FCD type IIb (after Bonferroni correction *p* < 0.005, paired *t*-test). Additionally, a significant decrease of the ReHo was detected at ipsilateral perilesional GM layer 1 compared to the CHRs in FCD type IIb. Notably, complete resection of functional perilesional GM alterations did not correlate with surgical outcomes. Compared to the contralateral homotopic GM, a decreased ALFF in the ipsilateral perilesional GM layer was detected in drug-responsive patients, whereas decreased ALFF in the ipsilateral perilesional GM layer 1–6 and decreased ReHo at ipsilateral perilesional GM layer 1 were observed in drug-resistant patients (after Bonferroni correction *p* < 0.005, paired *t*-test).

**Conclusion:**

Our findings indicate distinct functional profiles of perilesional GM based on FCD histological subtypes and ASMs’ response patterns. Importantly, our study illustrates that the identified functional alterations in perilesional GM may not provide sufficient evidence to determine the epileptogenic boundary required for surgical resection.

## 1 Introduction

Focal cortical dysplasia (FCD) is one of the foremost causes of drug-resistant focal epilepsy, which usually leads to epilepsy surgery, and is also one of the most common histological findings in respective epilepsy surgery (Harvey et al., 2008; Barkovich et al., 2012; Guerrini et al., 2015; Jin et al., 2016). FCD type II is the most common FCD in surgical patients, and is most frequently located in the frontal lobe (Blumcke et al., 2017). FCD type II can be sub-classified into FCD IIa (dysmorphic neurons) and FCD IIb (dysmorphic neurons with additional balloon cells) based on pathological classification (Najm et al., 2022). FCD IIb is more likely to have abnormal MRI compared to FCD type IIa (Studer et al., 2022; Macdonald-Laurs et al., 2023). Accurate detection and delineation of the epileptogenic boundaries of FCD are imperative, as favorable postsurgical seizure outcomes are typically related to the complete resection of FCD lesions, occasionally necessitating the excision of surrounding tissue (Jin et al., 2016; Willard et al., 2022).

Resting-state functional magnetic resonance imaging (rsfMRI) provides insights into intrinsic spontaneous neural activity (Lv et al., 2018). Utilizing the amplitude of low-frequency fluctuations (ALFF) and regional homogeneity (ReHo) has been instrumental in localizing the seizure onset zone (Chen et al., 2017; Tang et al., 2021) and exploring the potential epileptogenic boundary of Focal Cortical Dysplasia (FCD) (Hong et al., 2017; Gupta et al., 2018). As highlighted by Tang et al., ALFF exhibits a higher concordance rate than ReHo in localizing the seizure onset zone (Tang et al., 2021). Hong et al. demonstrated that ALFF and ReHo abnormalities extend beyond MRI-visible lesions, with distinct patterns in FCD type IIa compared to FCD type IIb (Hong et al., 2017). Despite these valuable insights into the functional alterations of perilesional gray matter (GM) and their potential relevance to epileptogenesis, a critical gap exists. No existing data explore the associations between the degree of resection of perilesional GM’s functional abnormalities and post-resection surgical outcomes. Thus, investigating the variations in functional alterations among different FCD type II subtypes and examining the correlation between complete resection of perilesional GM’s functional alterations and seizure outcomes is crucial and will significantly contribute to advancing our understanding of surgical management in this specific cohort of patients.

Several recent studies have reported that a minority of epileptic patients with FCD can be responsive to antiseizure medications (ASMs) for a sustained period of time or even life-long (Maynard et al., 2017; Chen et al., 2021; Cohen et al., 2022). Although studies have shown that several clinical factors may be associated with drug-resistant epilepsy, including younger age at seizure onset and failure of one ASM (Maynard et al., 2017; Cohen et al., 2022), the findings are inconsistent among studies. Our previous study explored the relationship between ASMs treatment response patterns and white matter microstructural alteration of FCD using diffusion tensor imaging (DTI), which found that compared to drug-responsive patients, drug-resistant patients exhibited more extensive perilesional white matter abnormalities (Jin et al., 2021). However, the associations between perilesional GM and ASMs responsive patterns remain unclear, and studies in perilesional GM of FCD are vital to further understanding of the underlying pathophysiology of FCD.

Thus, we aimed to (1) investigate the functional abnormalities of perilesional GM in FCD II subtypes and its correlation with the surgical outcome and (2) evaluate whether the functional profiles were specific to different ASMs treatment response patterns.

## 2 Materials and methods

### 2.1 Patient selection and classification of ASMs treatment outcomes

This retrospective study was approved by the institutional review boards of the Second Affiliated Hospital of Zhejiang University (SAHZU, 2022-0336/IR2022189). Written informed consent forms from all the enrolled subjects were obtained. We reviewed the consecutive medical charts of patients from the SAHZU between March 2013 and June 2021. The inclusion criteria were: (1) patients with MRI-identified FCD; (2) patients with histologically confirmed FCD; (3) patients who had at least 1 year of follow-up. Patients without rsfMRI and 3-dimensional (3D) T1 MRI were excluded.

The treatment outcomes of ASMs were evaluated every 3 months in this study. The outcomes were categorized into two groups: drug responsiveness and drug resistance. Drug responsiveness was defined as achieving seizure freedom for at least 1 year with ASMs (either as monotherapy or in combination with two or more drugs) at the last follow-up. Patients who did not meet the criteria for drug responsiveness were classified as drug-resistant. All patients with drug-resistant epilepsy underwent epilepsy surgery. The last visit before undergoing surgery was considered as the last follow-up visit. The surgical outcome was assessed using Engel’s classification scheme (Engel, 1993). Patients were classified as seizure-free if they maintained an Engel Class I score at their last follow-up. Postoperative clinical information was collected from follow-up clinical visits.

### 2.2 MRI acquisition

MRI scans were performed on a 3 T scanner (MR750, GE Healthcare) with the following sequences: three-dimensional (3D) T1-weighted fast spoiled gradient recalled echo (FSPGR) sequence (repetition time/echo time = 7.3/3.0 ms, inversion time = 450 ms, flip angle = 11°, matrix = 256 × 256, voxel size = 1 × 1 × 1mm^3^) and rsfMRI (repetition time/echo time = 2,000/30 ms, flip angle = 77°, matrix = 64 × 64, axial plane resolution = 3.75 × 3.75 mm^2^, slice thickness = 4 mm without slice gap, 38 slices and 205 volumes). Detailed parameters of two-dimensional T2W and fluid-attenuated inversion recovery (FLAIR) sequences were described in our previous study (Chen et al., 2021).

### 2.3 FCD masks

The indicative descriptions of FCD on MRI were blurring of gray-white matter junction, local cortical thickening, hyperintense signal on T2 or FLAIR sequences, the “transmantle sign” and abnormal sulcal or gyral pattern. The detailed information was published previously (Ding et al., 2018; Wang et al., 2019). FCD histological subtypes were classified according to the ILAE guidelines (Blumcke et al., 2011).

An experienced user (B.J.) independently manually segmented the FCD lesions based on 3D T1-weighted 3D images aided by FLAIR and other T2-weighted images using ITK-SNAP software, version 3.4.02^1^ (Yushkevich et al., 2006; Jin et al., 2018a). In order to account for variations in head sizes among individuals, the volume of FCD lesions was normalized by dividing the FCD lesion volume by the individual’s intracranial volume and then multiplying by 1,000,000 (normalized FCD lesion volume = FCD lesion volume × 1,000,000/individual intracranial volume). A log transformation was then applied to the normalized volumes to address the non-normal distribution of the volumes of FCD type II.

### 2.4 Image processing and analysis

**Figure 1:**
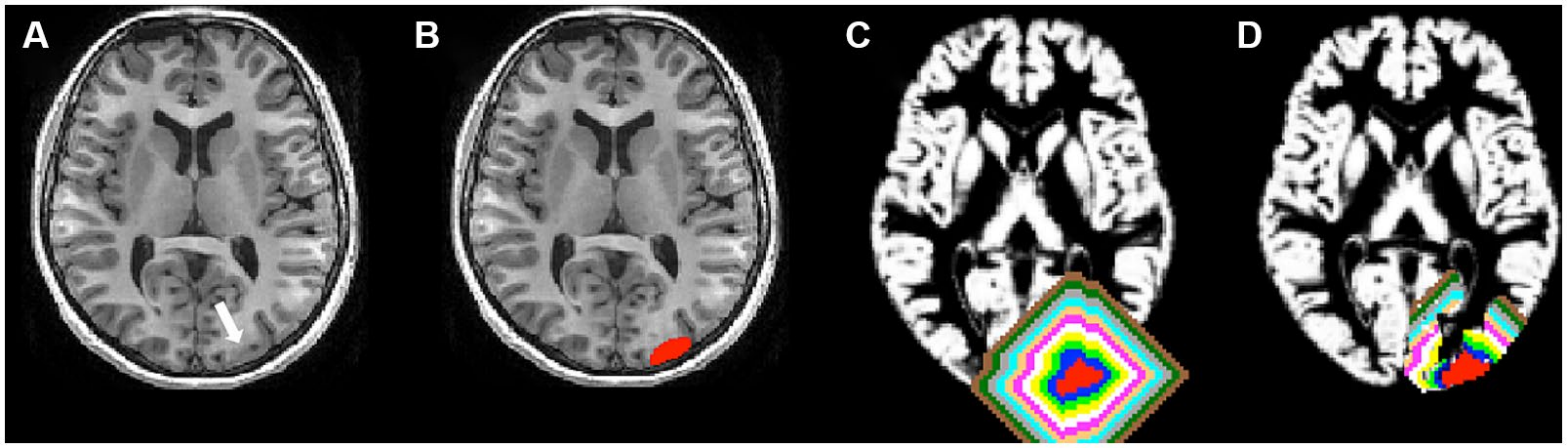
Lesion and perilesional gray matter (GM) layer masks. **(A)** The white arrow represented a lesion (FCD type IIb). **(B)** The red areas represented a lesion. **(C)** Each perilesional GM layer mask was dilated away from the FCD lesion by two voxels, for a total of 10 GM layers, respectively. The blue, green, yellow, white, pink, copper, cyan, gray, black green, and brown layers represent perilesional GM layer masks. The innermost layer adjoining the lesion was layer 1, and the outermost layer away from the lesion was layer 10. **(D)** All perilesional GM were cropped with the cortical mask, and regions extending to the contralateral hemisphere were removed. Then, the perilesional GM layers were created.

The 3D T1-weighted images were processed using FreeSurfer software (v.5.3.01) (Fischl et al., 2004). An experienced user (B.J.) independently reviewed the segmentation results of each subject for any errors before further analysis, and manual editing was performed for any inaccuracies. Hemispheric GM masks were created using FreeSurfer. The FCD lesions were nonlinearly registered to the Montreal Neurological Institute (MNI) space using FSL (v5.0.9^2^). Lesions were flipped onto the contralateral healthy hemisphere, forming contralateral homotopic regions (CHRs) in MNI space. Subsequently, individual-specific perilesional GM layer masks and contralateral homotopic GM layer masks were generated. The process involved sequential steps. Initially, the FCD lesion was dilated by two voxels (2 mm). Second, a new perilesional GM layer mask was derived by subtracting the FCD lesion from the dilated FCD lesion. Third, the new mask was intersected with the cortical mask, removing regions extending to the contralateral hemisphere and defining perilesional GM layer 1. Ultimately, 10 GM layers (20 mm) were created, with Layer 1 identified as the innermost and Layer 10 as the outermost, closest to the lesion. Contralateral homotopic lesions were derived from side-flipped original FCD masks using FSL. Each contralateral homotopic GM layer mask underwent a two-voxel (2 mm) dilation from the homotopic lesion, mirroring the steps taken for perilesional GM layer masks (Figure 1), resulting in uniformity in the creation of GM layers for both perilesional and contralateral homotopic regions.

The DPABI software (Yan et al., 2016) and Statistical Parametric Mapping (SPM12^3^) were accessed using the MATLAB platform (MathWorks) to preprocess the rsfMRI time series volume data. The first 10 volumes were discarded. Then, slice timing and motion correction were performed. Patients with head motion >3 mm of translation or 3 degrees of rotation were discarded. Then, the rsfMRI data were coregistered to the 3D T1 images and normalized into MNI space, followed by resampling the functional images to 3 × 3 × 3 mm^3^ isotropic voxels with a smoothing kernel of 6 mm (full width at half maximum). Also, signals from white matter and cerebrospinal fluid as well as Friston’s 24 motion parameters were regressed out as nuisance variables. Finally, linear detrending and temporal bandpass filtering (0.01–0.1 Hz) were performed.

Using DPABI software, ReHo analysis was performed. It was designed to measure the local synchronization of the times series of a certain voxel to its 26 nearest neighbors in a voxel-wise way, and it was calculated as Kendall’s coefficient of concordance (Zang et al., 2004). Each ReHo map was then divided by its average global ReHo of each patient for standardization purposes. Then, to reduce noise and residual differences, the data were smoothed with a Gaussian filter of 6 mm full width at half-maximum (FWHM).

The amplitude of low-frequency fluctuations (ALFF) was calculated in each voxel as the ratio of amplitudes in the low-frequency range (0.01–0.1 Hz) to that in the full-frequency range (0–0.25 Hz). To standardize data across subjects, the ALFF of each voxel was then divided by the global mean ALFF value of the individual. The perilesional GM layer mask and contralateral homotopic GM layer masks were applied to ALFF and ReHo maps individually.

The co-registration of preoperative MRI and postoperative MRI was performed using the SPM12 for surgical patients, and the resection region revealed by the postoperative MRI was compared with the abnormalities in the ALFF and ReHo maps. If abnormalities in ALFF and ReHo maps were entirely within the surgical resection area defined by postoperative MRI, we considered the surgical removal of identified functional alterations complete. Incomplete resection was defined by abnormalities in ALFF maps extending beyond the resection margins.

### 2.5 Statistical analysis

For between-group comparisons (drug-responsive and drug-resistant epilepsy; FCD type IIa and type IIb), if continuous variables (age, age at onset, duration of epilepsy) were normally distributed, 2-sample *t*-test was used. If not, the Mann–Whitney U test was used. Chi-square test was used for categorical variables (male, febrile seizures, family history, head trauma, CNS infection, perinatal adverse events, lesion lateralization, bottom-of-sulcus cortical dysplasia, focal to bilateral tonic–clonic seizure, lesion location, surgical outcome). For FCD lesion volumetric analysis, group differences were analyzed using analysis of covariance (ANCOVA) on age, gender. The paired *t*-test was used to determine differences in the ReHo and ALFF of perilesional GM and contralateral homotopic GM in two groups (drug-responsive and drug-resistant epilepsy; FCD type IIa and type IIb). A *p*-value of 0.005, obtained after applying Bonferroni corrections (0.05/10 = 0.005, Bonferroni corrections) was considered significant. Two-sample *t* test was used to compare group differences in the ReHo and ALFF of perilesional GM and contralateral homotopic GM. Bonferroni correction was further applied to correct for multiple comparisons. The statistical significance was set to *p* < 0.005 (0.05/10). The correlation between the volumetric data and the functional profiles of perilesional GM was assessed using Pearson correlation analysis. We used the chi-square test for surgical patients to assess the relationship between the abnormalities in the ALFF and ReHo maps and surgical outcomes. *p* < 0.05 was considered as statistical significance.

## 3 Results

### 3.1 Patient characteristics

A total of 39 patients were included, with 30 patients (76.9%) having drug-resistant epilepsy and nine patients classified as having drug-responsive epilepsy. All 30 patients with drug-resistant epilepsy underwent surgical resections, with 11 diagnosed with FCD type IIa and 19 with FCD type IIb. The mean age at seizure onset was 8.6 ± 6.4 years old, the mean age at the time of evaluation was 18.7 ± 8.6 years old, and the mean follow-up time was 29.8 ± 7.6 months. All 39 patients were MRI-positive, and the MRI features of these patients were highly indicative of FCD type II. FCD lesions were located in the following regions: frontal (28), posterior quadrant (5), insulo-opercular (4), and temporal (2). At the last follow-up visit, all nine patients with MRI-identified FCD met the criteria for drug-responsive epilepsy. Thirty patients with drug-resistant epilepsy underwent surgical resections.

### 3.2 ALFF and ReHo changes and FCD subtypes

**Figure 2:**
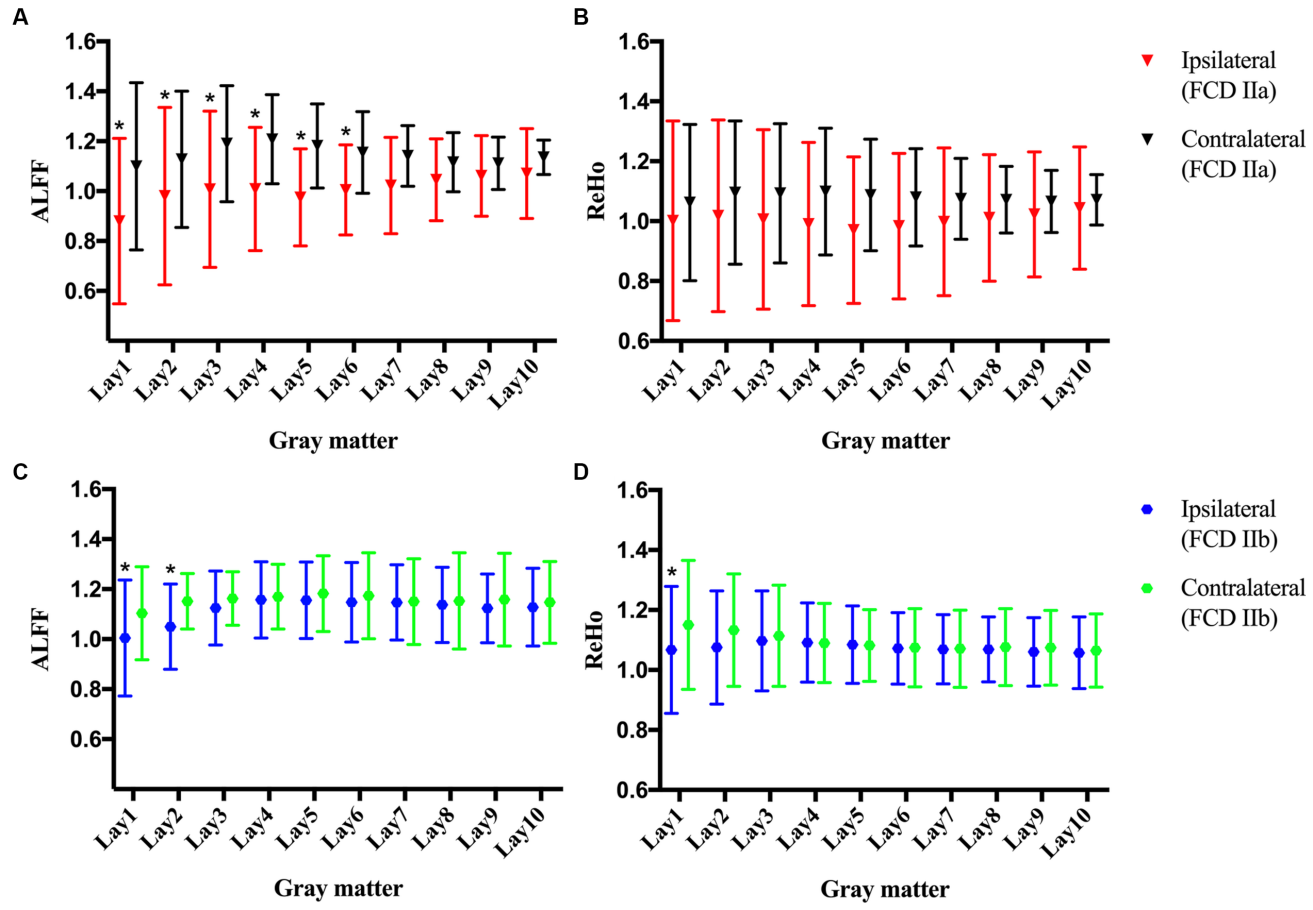
The amplitude of low-frequency fluctuations (ALFF) and regional homogeneity (ReHo) of the perilesional gray matter (GM) and contralateral homotopic regions in patients with FCD type IIa and FCD type IIb. **(A)** The ALFF of FCD type IIa. **(B)** The ReHo of FCD type IIa. **(C)** The ALFF of FCD type IIb. **(D)** The ReHo of FCD type IIb. Bonferroni correction was further applied to correct for multiple comparisons, *p < 0.005 represented the ipsilateral layers compared to the contralateral homotopic layers.

No statistically significant differences were observed in the clinical characteristics between the FCD type IIa group (*n* = 11) and the FCD type IIb group (*n* = 19, Table 1). Notably, FCD type IIa exhibited significant alterations in ALFF at ipsilateral perilesional GM layer 1–6 when compared to the contralateral homotopic GM layer 1–6 (after Bonferroni correction *p* < 0.005, Figure 2A). However, no significant differences were observed for the ReHo (Figure 2B). For FCD type IIb, a significant reduction in ALFF was noticed at ipsilateral perilesional GM layer 1–2 compared to the contralateral homotopic GM layer 1–2 (after Bonferroni correction *p* < 0.005, Figure 2C). And the ReHo was significantly decreased at ipsilateral perilesional GM layer 1, with respect to the contralateral homotopic GM layer 1 (after Bonferroni correction *p* < 0.005, Figure 2D). Compared to FCD type IIb, FCD type IIa showed a significant reduction on the ALFF at ipsilateral perilesional GM layer 4–6 (after Bonferroni correction *p* < 0.005). However, no statistical difference was noted in the ReHo of perilesional GM between FCD type IIa and FCD type IIb. Furthermore, there was no observed association between the ReHo or ALFF of perilesional GM and the volume of FCD type IIa or FCD type IIb.

**Table 1:** Comparison of clinical data between FCD Type IIa and Type IIb.

Clinical characteristics	FCD type IIa (*n* = 11)	FCD type IIb (*n* = 19)	*p* value
Male, *N* (%)^a^	5 (45.5%)	11 (57.9%)	0.707
Age at onset, Y^b^	8.0 ± 4.9	8.9 ± 6.0	0.677
Age, Y^b^	19.9 ± 7.3	19.7 ± 9.8	0.948
Duration of epilepsy, M^b^	142.9 ± 75.0	128.4 ± 104.3	0.694
Febrile seizures	0	0	
Family history	0	0	
Head trauma	0	0	
CNS infection	0	0	
Perinatal adverse events	0	1 (5.3%)	
Lesion lateralization (left)^a^	4(44.4%)	7 (36.7%)	0.979
BOSD^a^	6 (54.5%)	10 (52.6%)	1.000
FTBTCS^a^	6 (54.5%)	12 (63.2%)	0.712
**Lesion location**			
Frontal lobe	10 (90.9%)	12 (63.2%)	
Temporal lobe	0	1 (5.3%)	
Insular lobe	0	2 (10.5%)	
Posterior quadrant	1 (9.1%)	4 (21.0%)	
Seizure-free (Engel I)^a^	9 (81.8%)	17 (89.4%)	0.552

FCD, focal cortical dysplasia; CNS, central nervous system; BOSD, bottom-of-sulcus cortical dysplasia; FTBTCS, focal to bilateral tonic–clonic seizure.

a Chi-square test was used.

b 2-sample *t*-test was used.

### 3.3 ALFF and ReHo changes and surgical outcomes

**Figure 3:**
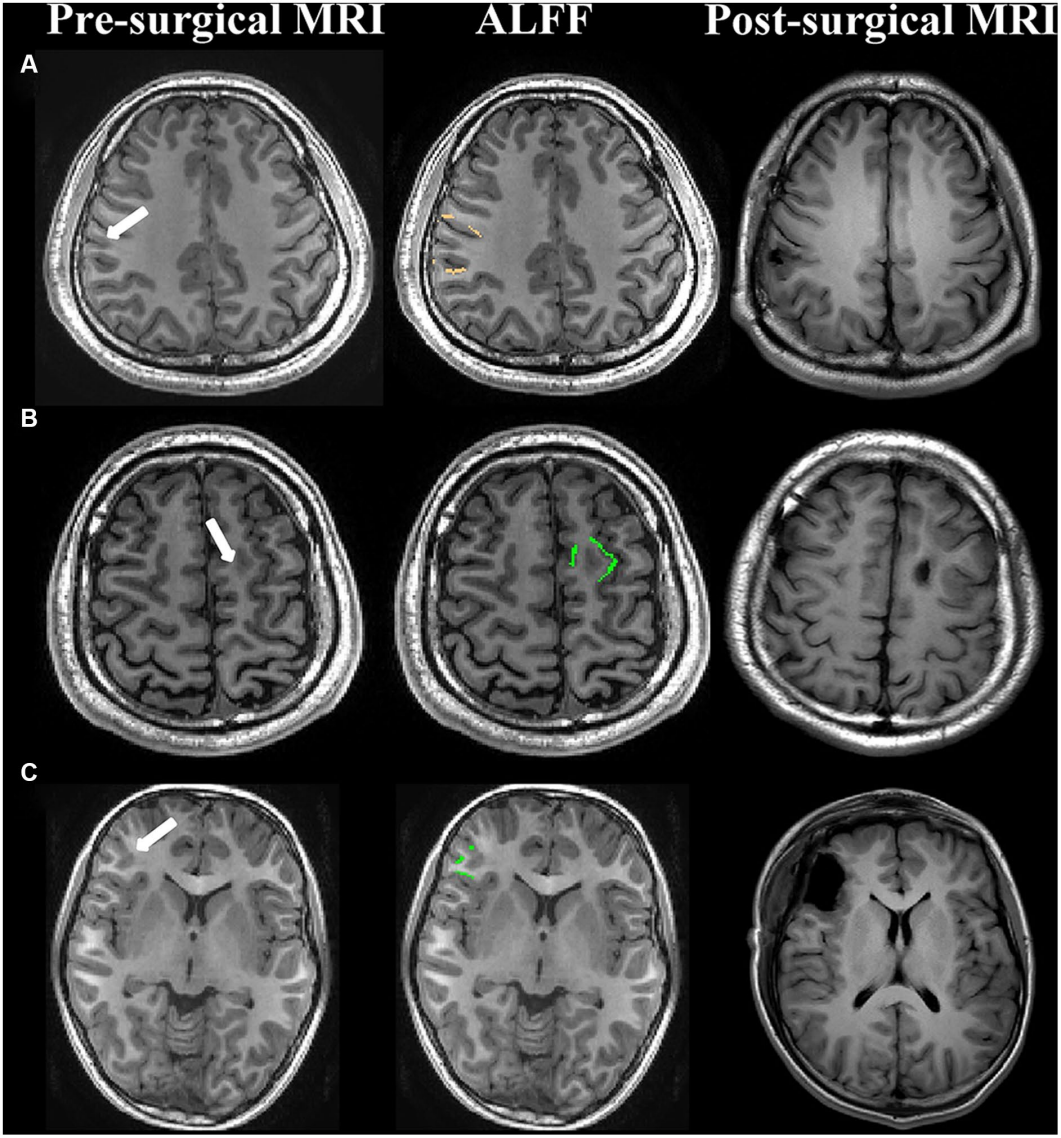
Examples of patients with abnormal ALFF maps within or outside the surgical regions. **(A)** FCD type IIa (seizure-free); **(B)** FCD type IIb (seizure-free); **(C)** FCD type IIb (seizure-free); First column: presurgical 3-dimensional T1-weighted images. The white arrow represented the lesion. Second column: the abnormal amplitude of low-frequency fluctuations maps in FCD type IIa and FCD type IIb were ipsilateral perilesional GM layer 6 (copper) and layer 2 (green), respectively. Third column: post-surgical MRI indicating the site and the extent of resection.

According to the above results, the abnormalities of ALFF maps in FCD type IIa and FCD type IIb were ipsilateral perilesional GM layer 6 and layer 2, respectively, and the abnormality of ReHo maps in FCD type IIb was ipsilateral perilesional GM layer 1. The abnormalities in the ALFF maps were outside the resection margins in all the patients with FCD type IIa, and 9 out of 11 patients became seizure-free. The abnormalities in the ALFF and ReHo maps were noted to be within the resection regions in 17 of 19 patients with FCD type IIb, and all 17 became seizure-free (Figure 3). No significant correlation was noted between the complete resection of abnormal ALFF and ReHo maps and surgical outcome in FCD type IIa or type IIb (*p* > 0.05, chi-square test).

### 3.4 ALFF and ReHo changes and drug treatment outcomes

**Figure 4:**
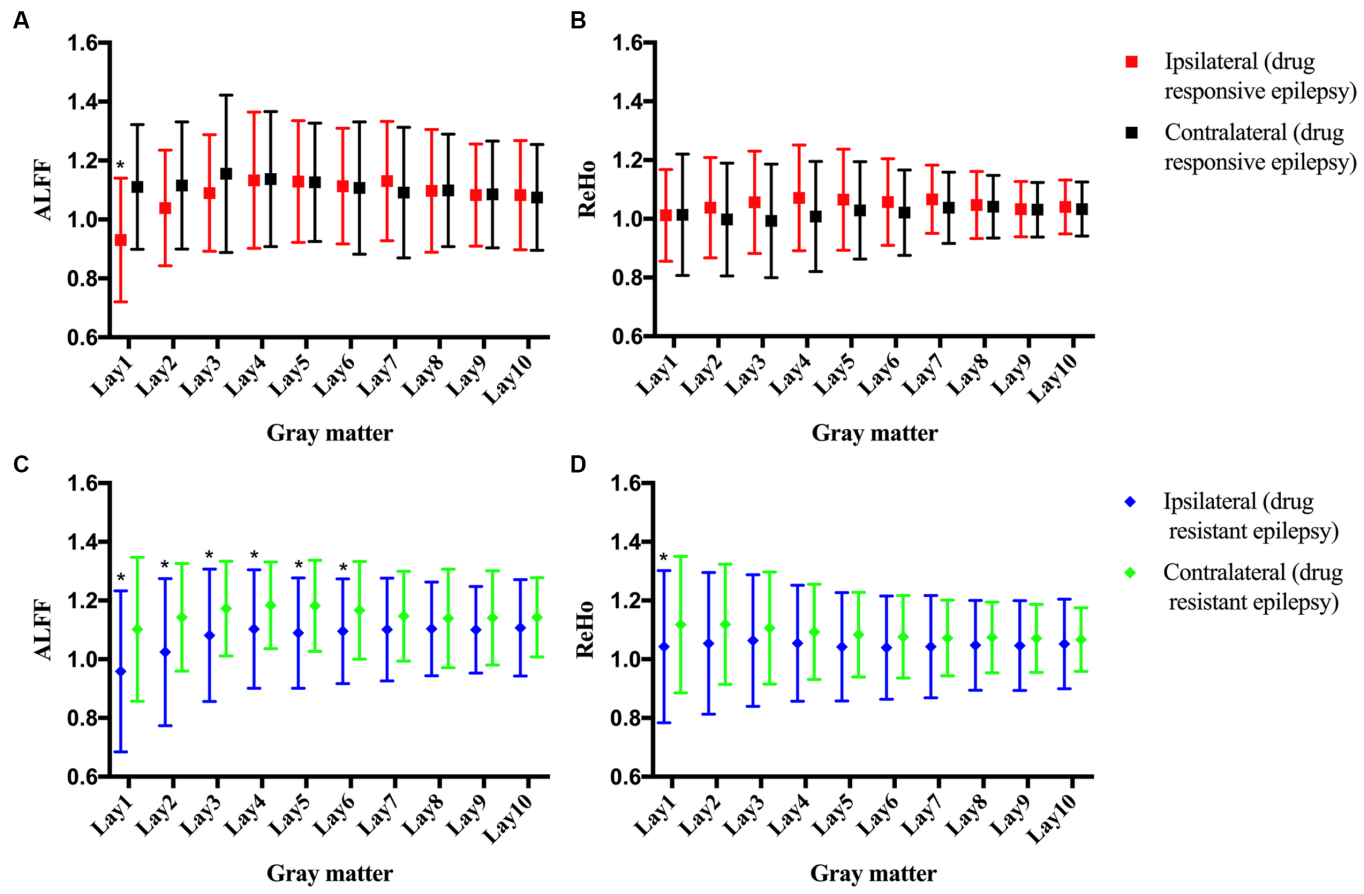
The amplitude of low-frequency fluctuations (ALFF) and regional homogeneity (ReHo) of the perilesional gray matter (GM) and contralateral homotopic region in patients with drug-resistant epilepsy and drug-responsive epilepsy. **(A)** The ALFF of drug-responsive epilepsy. **(B)** The ReHo of drug-responsive epilepsy. **(C)** The ALFF of drug-resistant epilepsy. **(D)** The ReHo of drug-resistant epilepsy. Bonferroni correction was further applied to correct for multiple comparisons, *p < 0.005 represented the ipsilateral layers compared to the contralateral homotopic layers.

No statistically significant differences in clinical characteristics were noted between the drug-responsive group (*n* = 9) and the drug-resistant group (*n* = 30), as detailed in Table 2. In patients with drug-responsive epilepsy, ALFF at ipsilateral perilesional gray matter GM layer 1 demonstrated a significantly lower corrected value compared to the contralateral homotopic GM layer 1 (after Bonferroni correction *p* < 0.005, Figure 4A). No significant differences were observed in the ReHo (Figure 4B). In drug-resistant epileptic patients, ALFF at ipsilateral perilesional GM layer 1 to 6 exhibited a significant decrease compared to the contralateral homotopic GM layer 1–6, respectively (after Bonferroni correction *p* < 0.005, Figure 4C). The ReHo demonstrated a substantial decrease at ipsilateral perilesional GM layer 1, relative to the contralateral homotopic GM layer 1 (after Bonferroni correction *p* < 0.005, Figure 4D). Compared to drug-responsive epilepsy, drug-resistant epilepsy showed a significant reduction in ALFF at ipsilateral perilesional GM layer 3–5 (after Bonferroni correction *p* < 0.005). No statistical difference was observed in the ReHo of perilesional GM between drug-resistant epilepsy and drug-responsive epilepsy.

**Table 2:** Comparison of clinical data in drug-responsive epilepsy vs. drug-resistant epilepsy.

Clinical characteristics	Drug-responsiveness (*n* = 9)	Drug-resistance (*n* = 30)	*p* value
Male, *N* (%)^a^	4 (44.4%)	16 (53.3%)	0.640
Age at onset, Y^b^	8.7 ± 7.4	8.6 ± 5.7	0.952
Age, Y^b^	15.4 ± 6.9	19.7 ± 8.9	0.189
Duration of epilepsy, M^b^	80.7 ± 56.7	133.7 ± 94.9	0.121
Febrile seizures	0	0	
Family history	0	0	
Head trauma	0	0	
CNS infection	0	0	
Perinatal adverse events	0	1 (3.3%)	
Lesion lateralization (left)^a^	4 (44.4%)	11 (36.7%)	0.674
BOSD^a^	5 (55.5%)	16 (53.3%)	0.907
FTBTCS^a^	3 (33.3%)	18 (60%)	0.255
**Lesion location**			
Frontal lobe	6 (66.7%)	22 (73.3%)	
Temporal lobe	1 (11.1%)	1 (3.3%)	
Insular lobe	2 (22.2%)	2 (6.7%)	
Posterior quadrant	0 (0%)	5 (16.7%)	
Seizure-free (Engel I)	–	26 (86.7%)	

FCD, focal cortical dysplasia; CNS, central nervous system; BOSD, bottom-of-sulcus cortical dysplasia; FTBTCS, focal to bilateral tonic–clonic seizure.

a Chi-square test was used.

b 2-sample *t*-test was used.

## 4 Discussion

The current study showed the functional profiles of perilesional GM in epileptic patients with FCD were different based on subtypes. FCD type IIa exhibited different functional profiles compared to FCD type IIb, suggesting that the severity and extent of perilesional GM alterations might relate to the histological subtypes. Furthermore, no significant correlation existed between complete resection of functional perilesional GM alterations and favorable surgical outcomes. Additionally, compared to patients with drug-responsive epilepsy, patients with drug-resistant epilepsy showed a more extensive functional perilesional GM abnormalities pattern.

In our cohort, we observed the ALFF alterations of FCD type IIa affecting a greater extent of perilesional GM layers than FCD type IIb. The ReHo alterations of FCD type IIa affected perilesional GM layers closer to the lesion than FCD type IIb. Notably, neither the abnormal ReHo nor ALFF of perilesional GM was associated with the volume of FCD type IIa or FCD type IIb. A recent mouse model of FCD indicated that epileptic seizures were initiated by the enhanced axonal connectivity between Ras Homolog Enriched in Brain 1 (RHEB) mutant neurons and distant neurons (Proietti Onori et al., 2021). Previous studies have highlighted a significant correlation between dysmorphic neurons and the onset of seizures and interictal discharges, while no such correlation was found with balloon cells (Boonyapisit et al., 2003; Cepeda et al., 2003; Rampp et al., 2021). Balloon cells were, however, correlated with white matter damage (Zucca et al., 2016; Zimmer et al., 2021). Furthermore, a marked reduction in dendritic spine density and quantitative alterations in synaptic markers were more pronounced in FCD type IIb compared to FCD type IIa (Rossini et al., 2021). In addition, a recent study utilizing connectome-based modeling demonstrated distinct functional connectivity patterns among different pathologic subtypes of FCD (Hong et al., 2019). FCD type IIb exhibited a decrease in local and whole-brain functional connectivity, whereas FCD type IIa showed an increase in both local and whole-brain functional connectivity (Hong et al., 2019). Hence, the observed disparity between the two FCD subtypes is not an unexpected finding. Besides, recognizing subtype-specific functional changes in FCD type II may enhance the efficacy of automatic lesion detection (Hong et al., 2017). Interestingly, there was no significant statistical difference in the ReHo and ALFF of perilesional GM and contralateral homotopic GM between FCD type IIa and FCD type IIb. These findings are likely due to the small sample in our cohort. Further studies with larger sample sizes are needed.

It has been shown that the complete removal of the cortex exhibiting dysplasia has a higher chance of achieving seizure freedom in patients with FCD (Jin et al., 2016; Willard et al., 2022). In clinical practice, structural MRI, PET, ictal SPECT, MEG, and DTI have been shown to play important roles in detecting subtle lesions and in localizing the seizure onset zone (Wang et al., 2015; Ding et al., 2018; Jin et al., 2018b; Ponisio et al., 2021; Guo et al., 2022; Gennari et al., 2023). Recent studies showed that rsfMRI might help localize the seizure onset zone (Boerwinkle et al., 2019; Tang et al., 2021). Interestingly, abnormalities are seen on rsfMRI in the surrounding cortex in FCD type II (Hong et al., 2017; Gupta et al., 2018), suggesting a smooth transition from the dysplastic lesion to the perilesional normal-appearing cortex. These results should raise a concern about the surgical extent of the dysplasia. In our cohort, complete resection of the abnormal ALFF and ReHo map was not correlated with seizure-free outcomes. Thus, our study illustrated that the extent of the functional perilesional GM alterations should not be used to determine the lesion borders for resection, and extra caution may be needed when using the functional profiles in the presurgical evaluation. Recently, a stereo-EEG study using partial directed coherence to compare dynamic connectivity changes under interictal, pre-ictal, and ictal states in FCD type II lesion and nonlesional surrounding cortex, the lesion showed increased connectivity, playing a leading role in generation and propagation, whereas nonlesional surrounding cortex showed lower connectivity, acting as a secondary generator of epileptiform activity (Varotto et al., 2012). Furthermore, one study reported that the spike and spike-high frequency oscillation cross rate reduced significantly over the gyral crown following the resection of the dysplasia sulcus in patients with bottom-of-sulcus dysplasia (BOSD) (Macdonald-Laurs et al., 2022). Thus, the abnormal perilesional normal-appearing cortex is the marker of altered local network connectivity (Macdonald-Laurs et al., 2022) rather than the marker of the resection margin. As the sample size in our study was small, further multi-center studies, including larger samples, are warranted to investigate whether all the GM abnormalities in the proximity of FCD lesions are essential to be resected in order to attain seizure freedom.

To our knowledge, this is the first study evaluating the relationship between ASM response patterns and the perilesional GM based on ALFF and ReHo in epileptic patients with FCD. We observed a decreased ALFF in the perilesional GM in both drug-resistant and drug-responsive groups. The abnormal functional perilesional GM was more restricted in patients with drug-responsive epilepsy than in patients with drug-resistant epilepsy. Decreased ReHo was observed in the drug-resistant group. These findings corroborate the hypothesis that in epileptic patients with FCD, the functional GM abnormalities extend beyond the MRI visible lesion. We also speculate that perilesional GM alterations might be one of the factors involved in developing drug resistance in epileptic patients with FCD. Those results may help unravel the underlying neuroanatomical character associated with drug resistance in epileptic patients with FCD, which could improve our ability to match patients to treatments.

Our study has limitations. Firstly, the cross-sectional nature of our study precludes the establishment of a causal link between the patterns of ASM response and GM abnormalities. Secondly, the sample size employed in our study was relatively modest, rendering it insufficient to facilitate a comprehensive analysis of the relationship between the specific location of FCD and the functional profiles of perilesional GM. Consequently, further research endeavors should be undertaken to elucidate these aspects more conclusively, employing prospective and longitudinal study designs with larger sample sizes.

## 5 Conclusion

Discovering the different functional profiles of perilesional GM is critical for further characterizing the affected areas and identifying the histological subtypes of FCD. Our study demonstrates that the functional perilesional GM alterations do not provide enough evidence to determine the epileptogenic boundary necessary for surgical treatment, and extra caution is needed to interpret the functional abnormality data in presurgical evaluation in drug-resistant epileptic patients with FCD. Additionally, our findings might help to unravel the underlying neuroanatomical character associated with drug resistance in epileptic patients with FCD and to improve the treatment strategy of epileptic patients with FCD in the near future.

## Data availability statement

The raw data supporting the conclusions of this article will be made available by the authors, without undue reservation.

## Ethics statement

The studies involving humans were approved by The Second Affiliated Hospital of Zhejiang University. The studies were conducted in accordance with the local legislation and institutional requirements. The participants provided their written informed consent to participate in this study.

## Author contributions

BJ: Conceptualization, Data curation, Formal analysis, Investigation, Methodology, Visualization, Writing – original draft, Writing – review & editing. JX: Conceptualization, Investigation, Validation, Writing – original draft. CW: Data curation, Formal analysis, Methodology, Writing – original draft. ShaW: Investigation, Validation, Writing – original draft. HL: Data curation, Methodology, Writing – original draft. CC: Formal analysis, Methodology, Writing – original draft. LY: Data curation, Formal analysis, Writing – original draft. CH: Data curation, Visualization, Writing – original draft. HC: Investigation, Writing – original draft. LZ: Conceptualization, Investigation, Writing – original draft. ShuW: Conceptualization, Data curation, Formal analysis, Investigation, Supervision, Visualization, Writing – original draft, Writing – review & editing. JW: Conceptualization, Data curation, Supervision, Visualization, Writing – original draft, Writing – review & editing. TA: Conceptualization, Writing – original draft, Writing – review & editing.

## Footnotes

^1^ http://www.itksnap.org

^2^ https://fsl.fmrib.ox.ac.uk/fsl

^3^ http://www.fil.ion.ucl.ac.uk/spm

## References

[ref1] Barkovich A. J. Guerrini R. Kuzniecky R. I. Jackson G. D. Dobyns W. B. (2012). A developmental and genetic classification for malformations of cortical development: update 2012. Brain 135, 1348–1369. doi: 10.1093/brain/aws019, PMID: 22427329 PMC3338922

[ref2] Blumcke I. Spreafico R. Haaker G. Coras R. Kobow K. Bien C. G.. (2017). Histopathological findings in brain tissue obtained during epilepsy surgery. N. Engl. J. Med. 377, 1648–1656. doi: 10.1056/NEJMoa1703784, PMID: 29069555

[ref3] Blumcke I. Thom M. Aronica E. Armstrong D. D. Vinters H. V. Palmini A.. (2011). The clinicopathologic spectrum of focal cortical dysplasias: a consensus classification proposed by an ad hoc task force of the ILAE diagnostic methods commission. Epilepsia 52, 158–174. doi: 10.1111/j.1528-1167.2010.02777.x, PMID: 21219302 PMC3058866

[ref4] Boerwinkle V. L. Cediel E. G. Mirea L. Williams K. Kerrigan J. F. Lam S.. (2019). Network-targeted approach and postoperative resting-state functional magnetic resonance imaging are associated with seizure outcome. Ann. Neurol. 86, 344–356. doi: 10.1002/ana.25547, PMID: 31294865

[ref5] Boonyapisit K. Najm I. Klem G. Ying Z. Burrier C. LaPresto E.. (2003). Epileptogenicity of focal malformations due to abnormal cortical development: direct electrocorticographic-histopathologic correlations. Epilepsia 44, 69–76. doi: 10.1046/j.1528-1157.2003.08102.x, PMID: 12581232

[ref6] Cepeda C. Hurst R. S. Flores-Hernandez J. Hernandez-Echeagaray E. Klapstein G. J. Boylan M. K.. (2003). Morphological and electrophysiological characterization of abnormal cell types in pediatric cortical dysplasia. J. Neurosci. Res. 72, 472–486. doi: 10.1002/jnr.10604, PMID: 12704809

[ref7] Chen Z. An Y. Zhao B. Yang W. Yu Q. Cai L.. (2017). The value of resting-state functional magnetic resonance imaging for detecting epileptogenic zones in patients with focal epilepsy. PLoS One 12:e0172094. doi: 10.1371/journal.pone.0172094, PMID: 28199371 PMC5310782

[ref8] Chen W. Jin B. Aung T. He C. Chen C. Wang S.. (2021). Response to antiseizure medications in epileptic patients with malformation of cortical development. Ther. Adv. Neurol. Disord. 14:17562864211050027. doi: 10.1177/17562864211050027, PMID: 34671424 PMC8521419

[ref9] Cohen N. T. Chang P. You X. Zhang A. Havens K. A. Oluigbo C. O.. (2022). Prevalence and risk factors for Pharmacoresistance in children with focal cortical dysplasia-related epilepsy. Neurology 99:e2006-2013. doi: 10.1212/WNL.0000000000201033, PMID: 35985831 PMC9651467

[ref10] Ding Y. Zhu Y. Jiang B. Zhou Y. Jin B. Hou H.. (2018). (18)F-FDG PET and high-resolution MRI co-registration for pre-surgical evaluation of patients with conventional MRI-negative refractory extra-temporal lobe epilepsy. Eur. J. Nucl. Med. Mol. Imaging 45, 1567–1572. doi: 10.1007/s00259-018-4017-0, PMID: 29671038

[ref002] Engel JJ. Rasmussen T.V.N.P. (1993). Outcome with respect to epileptic seizure. 2nd edn. New York: Raven Press 43:609–621.

[ref11] Fischl B. van der Kouwe A. Destrieux C. Halgren E. Segonne F. Salat D. H.. (2004). Automatically parcellating the human cerebral cortex. Cereb. Cortex 14, 11–22. doi: 10.1093/cercor/bhg087 14654453

[ref12] Gennari A. G. Cserpan D. Stefanos-Yakoub I. Kottke R. O'Gorman Tuura R. Ramantani G. (2023). Diffusion tensor imaging discriminates focal cortical dysplasia from normal brain parenchyma and differentiates between focal cortical dysplasia types. Insights Imaging 14:36. doi: 10.1186/s13244-023-01368-y, PMID: 36826756 PMC9958211

[ref13] Guerrini R. Duchowny M. Jayakar P. Krsek P. Kahane P. Tassi L.. (2015). Diagnostic methods and treatment options for focal cortical dysplasia. Epilepsia 56, 1669–1686. doi: 10.1111/epi.13200 26434565

[ref14] Guo K. Wang J. Cui B. Wang Y. Hou Y. Zhao G.. (2022). [(18)F]FDG PET/MRI and magnetoencephalography may improve presurgical localization of temporal lobe epilepsy. Eur. Radiol. 32, 3024–3034. doi: 10.1007/s00330-021-08336-4, PMID: 34651211

[ref15] Gupta L. Hofman P. A. M. Besseling R. M. H. Jansen J. F. A. Backes W. H. (2018). Abnormal blood oxygen level-dependent fluctuations in focal cortical dysplasia and the perilesional zone: initial findings. AJNR Am. J. Neuroradiol. 39, 1310–1315. doi: 10.3174/ajnr.A5684, PMID: 29794237 PMC7655428

[ref16] Harvey A. S. Cross J. H. Shinnar S. Mathern G. W. Taskforce I. P. E. S. S. (2008). Defining the spectrum of international practice in pediatric epilepsy surgery patients. Epilepsia 49, 146–155. doi: 10.1111/j.1528-1167.2007.01421.x, PMID: 18042232

[ref17] Hong S. J. Bernhardt B. C. Caldairou B. Hall J. A. Guiot M. C. Schrader D.. (2017). Multimodal MRI profiling of focal cortical dysplasia type II. Neurology 88, 734–742. doi: 10.1212/WNL.0000000000003632, PMID: 28130467 PMC5344077

[ref18] Hong S. J. Lee H. M. Gill R. Crane J. Sziklas V. Bernhardt B. C.. (2019). A connectome-based mechanistic model of focal cortical dysplasia. Brain 142, 688–699. doi: 10.1093/brain/awz009, PMID: 30726864 PMC6391612

[ref19] Jin B. Hu W. Ye L. Krishnan B. Aung T. Jones S. E.. (2018a). Small lesion size is associated with sleep-related epilepsy in focal cortical dysplasia type II. Front. Neurol. 9, –106. doi: 10.3389/fneur.2018.00106, PMID: 29541057 PMC5835765

[ref20] Jin B. Krishnan B. Adler S. Wagstyl K. Hu W. Jones S.. (2018b). Automated detection of focal cortical dysplasia type II with surface-based magnetic resonance imaging postprocessing and machine learning. Epilepsia 59, 982–992. doi: 10.1111/epi.14064, PMID: 29637549 PMC5934310

[ref21] Jin B. Lv Z. Chen W. Wang C. Aung T. Chen W.. (2021). Perilesional white matter integrity in drug-resistant epilepsy related to focal cortical dysplasia. Seizure 91, 484–489. doi: 10.1016/j.seizure.2021.07.027, PMID: 34343860

[ref22] Jin B. Wang J. Zhou J. Wang S. Guan Y. Chen S. (2016). A longitudinal study of surgical outcome of pharmacoresistant epilepsy caused by focal cortical dysplasia. J. Neurol. 263, 2403–2410. doi: 10.1007/s00415-016-8274-1, PMID: 27632178

[ref001] Lv H. Wang Z. Tong E. Williams L.M. Zaharchuk G. Zeineh M.. (2018). Resting-State Functional MRI: Everything That Nonexperts Have Always Wanted to Know. AJNR Am J Neuroradiol. 39, 1390–1399. doi: 10.3174/ajnr.A5527 29348136 PMC6051935

[ref23] Macdonald-Laurs E. Warren A. E. L. Francis P. Mandelstam S. A. Lee W. S. Coleman M.. (2023). The clinical, imaging, pathological and genetic landscape of bottom-of-sulcus dysplasia. Brain:awad379. doi: 10.1093/brain/awad379, PMID: 37939785

[ref24] Macdonald-Laurs E. Warren A. E. L. Lee W. S. Yang J. Y. MacGregor D. Lockhart P. J.. (2022). Intrinsic and secondary epileptogenicity in focal cortical dysplasia type II. Epilepsia 64, 348–363. doi: 10.1111/epi.17495, PMID: 36527426 PMC10952144

[ref25] Maynard L. M. Leach J. L. Horn P. S. Spaeth C. G. Mangano F. T. Holland K. D.. (2017). Epilepsy prevalence and severity predictors in MRI-identified focal cortical dysplasia. Epilepsy Res. 132, 41–49. doi: 10.1016/j.eplepsyres.2017.03.001 28288357

[ref26] Najm I. Lal D. Alonso Vanegas M. Cendes F. Lopes-Cendes I. Palmini A.. (2022). The ILAE consensus classification of focal cortical dysplasia: An update proposed by an ad hoc task force of the ILAE diagnostic methods commission. Epilepsia 63, 1899–1919. doi: 10.1111/epi.17301 35706131 PMC9545778

[ref27] Ponisio M. R. Zempel J. M. Day B. K. Eisenman L. N. Miller-Thomas M. M. Smyth M. D.. (2021). The role of SPECT and PET in epilepsy. AJR Am. J. Roentgenol. 216, 759–768. doi: 10.2214/AJR.20.23336, PMID: 33474983

[ref28] Proietti Onori M. Koene L. M. C. Schafer C. B. Nellist M. de Brito van Velze M. Gao Z.. (2021). RHEB/mTOR hyperactivity causes cortical malformations and epileptic seizures through increased axonal connectivity. PLoS Biol. 19:e3001279. doi: 10.1371/journal.pbio.3001279, PMID: 34038402 PMC8186814

[ref29] Rampp S. Rossler K. Hamer H. Illek M. Buchfelder M. Doerfler A.. (2021). Dysmorphic neurons as cellular source for phase-amplitude coupling in focal cortical dysplasia type II. Clin. Neurophysiol. 132, 782–792. doi: 10.1016/j.clinph.2021.01.004, PMID: 33571886

[ref30] Rossini L. De Santis D. Mauceri R. R. Tesoriero C. Bentivoglio M. Maderna E.. (2021). Dendritic pathology, spine loss and synaptic reorganization in human cortex from epilepsy patients. Brain 144, 251–265. doi: 10.1093/brain/awaa387 33221837

[ref31] Studer M. Rossini L. Spreafico R. Pelliccia V. Tassi L. de Curtis M.. (2022). Why are type II focal cortical dysplasias frequently located at the bottom of sulcus? A neurodevelopmental hypothesis. Epilepsia 63, 2716–2721. doi: 10.1111/epi.17386 35932101

[ref32] Tang Y. Choi J. Y. Alexopoulos A. Murakami H. Daifu-Kobayashi M. Zhou Q.. (2021). Individual localization value of resting-state fMRI in epilepsy presurgical evaluation: a combined study with stereo-EEG. Clin. Neurophysiol. 132, 3197–3206. doi: 10.1016/j.clinph.2021.07.028, PMID: 34538574 PMC8629929

[ref33] Varotto G. Tassi L. Franceschetti S. Spreafico R. Panzica F. (2012). Epileptogenic networks of type II focal cortical dysplasia: a stereo-EEG study. NeuroImage 61, 591–598. doi: 10.1016/j.neuroimage.2012.03.090, PMID: 22510255

[ref34] Wang Z. I. Jones S. E. Jaisani Z. Najm I. M. Prayson R. A. Burgess R. C.. (2015). Voxel-based morphometric magnetic resonance imaging (MRI) postprocessing in MRI-negative epilepsies. Ann. Neurol. 77, 1060–1075. doi: 10.1002/ana.24407, PMID: 25807928 PMC4447617

[ref35] Wang S. Tang Y. Aung T. Chen C. Katagiri M. Jones S. E.. (2019). Multimodal noninvasive evaluation in MRI-negative operculoinsular epilepsy. J. Neurosurg. 132, 1, 1334–11. doi: 10.3171/2018.12.JNS182746, PMID: 30978689

[ref36] Willard A. Antonic-Baker A. Chen Z. O'Brien T. J. Kwan P. Perucca P. (2022). Seizure outcome after surgery for MRI-diagnosed focal cortical dysplasia: a systematic review and Meta-analysis. Neurology 98, e236–e248. doi: 10.1212/WNL.0000000000013066, PMID: 34893558

[ref37] Yan C. G. Wang X. D. Zuo X. N. Zang Y. F. (2016). DPABI: Data Processing & Analysis for (resting-state) brain imaging. Neuroinformatics 14, 339–351. doi: 10.1007/s12021-016-9299-4 27075850

[ref38] Yushkevich P. A. Piven J. Hazlett H. C. Smith R. G. Ho S. Gee J. C.. (2006). User-guided 3D active contour segmentation of anatomical structures: significantly improved efficiency and reliability. NeuroImage 31, 1116–1128. doi: 10.1016/j.neuroimage.2006.01.015, PMID: 16545965

[ref39] Zang Y. Jiang T. Lu Y. He Y. Tian L. (2004). Regional homogeneity approach to fMRI data analysis. NeuroImage 22, 394–400. doi: 10.1016/j.neuroimage.2003.12.030 15110032

[ref40] Zimmer T. S. Broekaart D. W. M. Luinenburg M. Mijnsbergen C. Anink J. J. Sim N. S.. (2021). Balloon cells promote immune system activation in focal cortical dysplasia type 2b. Neuropathol. Appl. Neurobiol. 47, 826–839. doi: 10.1111/nan.12736, PMID: 34003514 PMC8518746

[ref41] Zucca I. Milesi G. Medici V. Tassi L. Didato G. Cardinale F.. (2016). Type II focal cortical dysplasia: *ex vivo* 7T magnetic resonance imaging abnormalities and histopathological comparisons. Ann. Neurol. 79, 42–58. doi: 10.1002/ana.24541, PMID: 26448158

